# Genetic Dissection of Snow Mold Tolerance in US Pacific Northwest Winter Wheat Through Genome-Wide Association Study and Genomic Selection

**DOI:** 10.3389/fpls.2019.01337

**Published:** 2019-10-29

**Authors:** Dennis Lozada, Jayfred V. Godoy, Timothy D. Murray, Brian P. Ward, Arron H. Carter

**Affiliations:** ^1^Department of Crop and Soil Sciences, Washington State University, Pullman, WA, United States; ^2^Department of Plant Pathology, Washington State University, Pullman, WA, United States; ^3^USDA-ARS Plant Science Research Unit, Raleigh, NC, United States

**Keywords:** genome-wide association study, genomic best linear unbiased prediction, genomic selection, reproducing kernel Hilbert space, ridge regression best linear unbiased prediction, snow mold tolerance, winter wheat

## Abstract

Snow mold is a yield-limiting disease of wheat in the Pacific Northwest (PNW) region of the US, where there is prolonged snow cover. The objectives of this study were to identify genomic regions associated with snow mold tolerance in a diverse panel of PNW winter wheat lines in a genome-wide association study (GWAS) and to evaluate the usefulness of genomic selection (GS) for snow mold tolerance. An association mapping panel (AMP; *N* = 458 lines) was planted in Mansfield and Waterville, WA in 2017 and 2018 and genotyped using the Illumina^®^ 90K single nucleotide polymorphism (SNP) array. GWAS identified 100 significant markers across 17 chromosomes, where SNPs on chromosomes 5A and 5B coincided with major freezing tolerance and vernalization loci. Increased number of favorable alleles was related to improved snow mold tolerance. Independent predictions using the AMP as a training population (TP) to predict snow mold tolerance of breeding lines evaluated between 2015 and 2018 resulted in a mean accuracy of 0.36 across models and marker sets. Modeling nonadditive effects improved accuracy even in the absence of a close genetic relatedness between the TP and selection candidates. Selecting lines based on genomic estimated breeding values and tolerance scores resulted in a 24% increase in tolerance. The identified genomic regions associated with snow mold tolerance demonstrated the genetic complexity of this trait and the difficulty in selecting tolerant lines using markers. GS was validated and showed potential for use in PNW winter wheat for selecting on complex traits such tolerance to snow mold.

## Introduction

Snow mold is a disease affecting wheat grown in the Pacific Northwest (PNW) region of the US, where plants are exposed to prolonged (>100 days) snow cover. This prolonged winter condition provides a favorable environment, allowing the pathogens causing the disease to grow and infect plants under the snow (Bruehl and Cunfer, 1971). In the state of Washington, there are four different snow mold diseases, all caused by soil-borne fungi or fungal-like organisms: pink snow mold (*Microdochium* [*Fusarium*] *nivale*), speckled snow mold (*Typhula idahoensis, T. ishikariensis, T. incarnata*), snow scald (*Myriosclerotinia borealis*), and snow rot (*Pythium iwayami* and *P. okanoganense*) (Murray et al., 1999). Following snowmelt, plants with pink snow mold have a whitish fungal growth (that eventually turns into a salmon color; hence the name pink snow mold) in their leaves, whereas leaves of plants with speckled snow mold are covered with a whitish gray fungal growth (Murray et al., 1999).

Although cultural practices such as seeding date, fertilizer application, and residue management can be used to manage snow mold in wheat, breeding for disease tolerance was suggested as the primary method of control for snow mold (Murray et al., 1999). There are several challenges associated with breeding for snow mold tolerance in winter wheat. Screening is difficult because snow cover is highly variable within fields with some areas not having enough snow cover for the disease to develop and other areas having drifts that cause extended snow cover and severe disease. The amount of snow also varies from year to year and, consequently, disease pressure is highly variable across growing seasons. Without the necessary conditions for disease development, it is difficult to identify tolerant lines. “Winter warming” has caused changes in the total PNW area covered with snow (Nolin and Daly, 2006), which also affects the distribution and severity of snow mold. Furthermore, there are a limited number of wheat and related species showing tolerance to the disease (Bruehl, 1982; Iriki et al., 2001). The use of molecular breeding approaches to identify tolerant lines and to better understand the genetic basis of the disease is therefore relevant for improving snow mold tolerance in current winter wheat lines.

In recent years, tools such as genome-wide association study (GWAS) have been used to examine the genetic architecture underlying important traits in wheat, such as grain yield (Neumann et al., 2011; Sukumaran et al., 2015; Lozada et al., 2017), disease resistance, including spot blotch (Ayana et al., 2018), Fusarium head blight (Arruda et al., 2016), and stripe rust (Naruoka et al., 2015; Liu et al., 2018), and heading date, plant height, and thousand grain weight (Zanke et al., 2014; Zanke et al., 2015; Godoy et al., 2018), among others. GWAS uses the concept of linkage disequilibrium, i.e., the nonrandom cosegregation of alleles at multiple loci, to identify significant marker-trait associations in natural populations (Flint-Garcia et al., 2003; Breseghello and Sorrells, 2006). In contrast to traditional biparental traditional biparental quantitative trait loci (QTL) mapping, GWAS has a higher mapping resolution due to the large number of recombination events observed in the diverse populations used (Zhu et al., 2008; Myles et al., 2009). Furthermore, GWAS allows for genetic survey of much larger populations and avoids the time needed for mapping population development (as in the case of biparental QTL analyses) (Neumann et al., 2011).

GWAS, nevertheless, cannot identify variants with small effects (Korte and Farlow, 2013), and this becomes a limitation as many traits are affected by multiple loci with minor effects. Genomic selection (GS) overcomes this limitation by using genome-wide marker data to predict the breeding values of individuals (Meuwissen et al., 2001). In contrast to association mapping, GS does not test for significance of markers but instead considers all marker information to train a model and predict breeding values (BV) of individuals (Jiang et al., 2015; Crossa et al., 2017). It assumes that the BV can be estimated purely based on molecular marker data (Qian et al., 2017). A model is first trained using a training panel (whose genotype and phenotype are known) and then used for prediction of BV in test population with marker data but no phenotypic data (Crossa et al., 2017). These values are ultimately used in breeding programs for the selection of promising genotypes (Werner et al., 2018).

Kruse et al. (2017) previously identified important QTL on chromosomes 5A and 6B associated with tolerance to snow mold in a biparental population derived from the cross between winter wheat varieties “Eltan” (Peterson et al., 1991) and “Finch” (Garland-Campbell et al., 2005). Currently, there are no known SNP markers associated with snow mold tolerance in PNW winter wheat lines identified through GWAS. The objectives of the current study were to i) identify genomic regions associated with tolerance to snow mold in a diverse panel of winter wheat lines adapted to the PNW and ii) evaluate the accuracy of GS for snow mold tolerance using breeding lines from the Washington State University (WSU) Winter Wheat Breeding and Genetics Program for independent predictions. The efficiency of phenotypic selection (PS), GS, and combining PS with GS was also evaluated for tolerance to snow mold in terms of response to selection.

## Materials and Methods

### Plant Material

An association mapping panel (AMP) consisting of 458 advanced soft winter wheat breeding lines adapted to the US Pacific Northwest (PNW) region was used for GWAS and as a training population (TP) for GS. This population consisted of club (172 lines; 37.6%) and common (286; 62.4%) wheat. The AMP was previously characterized for different traits including stripe rust resistance (Naruoka et al., 2015; Liu et al., 2018), eyespot resistance (Lewien et al., 2018), end-use quality traits (Jernigan et al., 2018), preharvest sprouting tolerance, and low falling number (Martinez et al., 2018). Additionally, 295 winter wheat breeding lines from the Washington State University (WSU) Winter Wheat Breeding and Genetics Program evaluated for snow mold tolerance between 2015 and 2018 were used as selection candidates (TST lines) for independent validations. Breeding lines range from F_4:6_ to F_4:9_ selections. Six varieties, “Bruehl” (Jones et al., 2001), Eltan, “Madsen” (Allan et al., 1989), “Masami” (Jones et al., 2006), “Stephens” (Kronstad et al., 1978), and “Xerpha” (Jones et al., 2010) were common to both the TP and TST populations.

### Field Evaluation for Snow Mold Tolerance

The AMP and breeding lines were planted in Mansfield (MAN) and Waterville (WAT), WA for 2017-2018 (for the AMP) and 2015-2018 seasons (for the TST lines). Tolerance to snow mold was evaluated by rating the lines within a few days of snow melt and one month later using a 0 (completely dead, with abundance of snow mold) to 10 (thriving, no snow mold) scale. Each rating was considered a unique trait. Varieties “Eltan” (Peterson et al., 1991) and “Finch” (Garland-Campbell et al., 2005) with moderate tolerance and susceptibility to snow mold, respectively, were included in the AMP. The TST lines were evaluated between 2015 and 2018, where snow mold ratings were taken only in WAT in 2015, and both in MAN and WAT for 2015-2018. In 2015, the Mansfield location had a significant amount of erosion caused by spring runoff, and thus snow mold scores were not collected at this location. Disease scoring commenced as soon as snow had melted from the plot location and it was dry enough to enter the field.

The AMP was planted in late August each year using a custom built deep-furrow four-row planter spaced 30 cm apart. Entries were planted in two of the four rows as paired rows, so that each plot contained two entries of the AMP, one in the left-side two rows and one in the right-side two rows. The trial was planted as an alpha-lattice design, and two replications were planted. In between each of the incomplete blocks, a plot consisting of one row of Eltan (moderately tolerant; Peterson et al., 1991), “Otto” (tolerant; Carter et al., 2013), “Bruehl” (moderately tolerant; Jones et al., 2001), and “Puma” (susceptible, Carter et al., 2014) was planted as check varieties. Plots were 1.5 meter in length, each variety covered an area of 1 m^2^, planted at 100 plants per m^2^. PROC GLM in SAS v 9.4 (SAS Institute, 2016) was used to analyze the spatial variation among the repeating check cultivars. In both years and across locations, no significant differences were found in traits values of check cultivars spaced throughout the trial, indicating uniformity of snow mold infection, and thus no spatial adjustment was conducted on the lines within the AMP.

The TST lines were planted at the same time as the AMP and in a similar fashion, except the TST lines were planted using a randomized-complete block design with three replications. The same four row check plot was used to evaluate spatial variation and planted every 10^th^ plot within the TST lines. Analysis of these plots within the TST lines again found no significant differences between checks within a given location-year, and thus no spatial adjustment was conducted on the TST lines.

### Trait Heritability

Pearson correlations across environments were calculated using JMP v. 11.0.0 (JMP^®^ SAS Institute, 2013). Broad sense heritability (*H^2^*) was computed by considering genotype, environment, and genotype by environment as random effects and estimating their variance components through PROC mixed in SAS v 9.4 using the formula $H^{2}=\;\frac{\sigma_{G}^{2}}{\sigma_{G\;}^{2}+\;\;\sigma_{\frac{\mathrm{GEI}}{e}}^{2}\;+\;\;\sigma_{\frac{E}{\mathrm{er}}}^{2}\;\;\;}$, where $\sigma_{G\;}^{2}$, $\sigma_{\mathrm{GEI}\;}^{2}$, and $\sigma_{E\;}^{2}$ are variances due to genotype, genotype-by- environment, and error, respectively; *e* and *r* are the number of environments and replications, respectively.

### SNP Marker Genotyping, Genetic Diversity, and Genome-Wide Association Analyses

The AMP was genotyped using the Illumina 90K SNP assays (Wang et al., 2014) at the USDA-ARS Northern Genotyping Laboratory, Fargo, ND. Allele calling and curation was done using GenomeStudio^®^ v. 2011.1 (Illumina, United States). The SNP marker information has been deposited in the GrainGenes Database, reference PBJ-12-787. After filtering for SNPs with minor allele frequency (MAF) > 0.05 and removing markers with >20% missing data, 15,229 high-quality SNPs remained for GWAS, of which 12,681 SNPs (83.3%) have been mapped across all chromosomes. SNP positions (in cM) were based on the consensus genetic linkage map for hexaploid wheat reported by Wang et al. (2014).

Genetic diversity was evaluated by calculating Rogers distances and population differentiation coefficients (*F_st_*) using SNP marker data. Population structure within the AMP and breeding lines was assessed using principal components analysis (PCA), where the second principal component (PC2) was plotted against PC1. All analyses were performed in JMP Genomics v 8.1 (JMP^®^ SAS Institute, 2013).

Association mapping was conducted using the Fixed and Random Model Circulating Probability Unification (FarmCPU) package (Liu et al., 2016) implemented in R, where PCs calculated using GAPIT (Lipka et al., 2012) were fitted in the model as fixed effect. Four different GWAS models were tested based on the presence of different number of PCs- PC0, PC1, PC2, and PC3, corresponding to the inclusion of 0, 1, 2, and 3 PCs, respectively. Significant associations were based on a Benjamini-Hochberg false discovery rate (FDR; Benjamini and Hochberg, 1995) of 0.05. Phenotypic variation explained by each SNP was calculated by fitting genotype and trait data on a stepwise regression model and calculating the difference between the effects when all significant markers are present and when an SNP is removed from the model in JMP v. 11.0.0. A significant SNP was considered stable when it was identified in at least two environments and/or GWAS models. Best linear unbiased prediction (BLUP) values for each of the lines were calculated by combining individual locations across years using restricted maximum estimate likelihood (REML) method in JMP v. 11.0.0, where genotype, environment, and genotype by environment interactions were considered random effects. These resulted in a total of seven BLUP datasets used for association mapping. GWAS models were compared by examining the deviations observed in quantile-quantile (QQ) plots; the model with the least number of deviations observed was regarded as the most reliable in identifying marker-trait associations.

To test the effect of pyramiding favorable alleles on snow mold tolerance, BLUP scores were plotted against increasing numbers of favorable alleles for the 10 most significant SNPs identified through GWAS. Correlation coefficient between BLUP and the number of favorable alleles was calculated in JMP v. 11.0.0 Association of snow mold tolerance with haplotypes of significant SNPs was determined using the PROC HAPLOTYPE procedure in SAS v 9.4, whereas linkage disequilibrium (LD) between marker pairs was evaluated using JMP Genomics v.8.1. Significant marker pairs in the same chromosome were considered in LD at *P* < 0.05.

### Genomic Selection

Accuracy of GS for tolerance to snow mold was evaluated by performing independent predictions using the AMP as TP, and WSU winter wheat breeding lines evaluated between 2015 and 2018 as test populations (TST2015, TST2016, TST2017, and TST2018). The AMP and the breeding lines were genotyped using genotyping by sequencing (GBS) following a *PstI-MspI* restriction enzyme double digest (Poland et al., 2012). After filtering for markers with MAF > 0.05 and quality control, 16,233 SNP markers common to both TP and validation sets remained for analyses. To test the effect of marker number on the accuracy of GS, two other marker sets (MS) in addition to the whole genotype marker data (MS1) were used for predictions: a set consisting of significant markers (*P* < 0.05) identified through GWAS (MS2) using only the training set (AMP) to prevent “inside trading” effects, observed previously to cause overestimated accuracies (Arruda et al., 2016); and a set of LD-tag selected SNPs (MS3), derived from a grouping algorithm developed by Carlson et al. (2004) implemented in JMP Genomics v. 8.1.

GS was conducted using three different models: Ridge regression best linear unbiased prediction (RRBLUP) through the package “rrBLUP” (Endelman, 2011), Genomic best linear unbiased predictions (GBLUP), and Reproducing Kernel Hilbert Spaces (RKHS), through the Bayesian generalized linear regression (BGLR) package (Pérez and de los Campos, 2014). GBLUP and RKHS models were fitted using 12,000 iterations and 2,000 burn-ins. All GS models were implemented in R statistical software (R Development Core Team, 2018).

The basic model for RRBLUP is y = **WGu** + ε, where **u** represents the vector of marker effects, **G** is genotype matrix under an additive model, and **W** is a design matrix relating lines to phenotypes (observations) (Endelman, 2011). RRBLUP shrinks marker effects toward zero and assumes that markers have equal variances (Heffner et al., 2011). GBLUP, on the other hand, uses a genomic relationship matrix derived from markers to calculate an individual’s genetic merit (Clark and van der Werf, 2013). The model for GBLUP is in the form y = **X**b + **Z**g + e, where y is a vector of phenotypes, **X** is a design matrix of fixed effects, b is a vector of fixed effects, **Z** is design matrix of genetic values, g is a vector of additive genetic effects, and e is a vector of random effects (Clark and van der Werf, 2013).

RRBLUP and GBLUP are mathematically equivalent (Habier et al., 2007) but are still considered to be different approaches for estimating GEBV. RRBLUP estimates marker effects using linear and penalized parameters, whereas GBLUP does not depend on marker effect estimation for calculating breeding values (Tan et al., 2017). Bayesian RKHS models can be represented as y = 1µ + **u** + ε with *p*(µ, u, ε)} N (u|0,**K**σ^2^_u_)N(ε|0,Iσ^2^_ε_), where µ is the intercept, u is vector of random effects, ε is a vector of residuals, σ^2^_ε_ is residual variance, and **K** is a Kernel matrix calculated as the squared-Euclidian distance between genotypes (Pérez and de los Campos, 2014).

### Comparison Between Genomic Estimated Breeding Values and Tolerance Scores

To examine the relationship of breeding values with snow mold tolerance scores for lines advanced in the following year, GEBV of lines from a single year was compared to their mean tolerance scores observed in the succeeding year (i.e., 2015 GEBV compared to 2016 snow mold tolerance (SMT) scores; 2016 GEBV to 2017 SMT; and 2017 GEBV to 2018 SMT). A ridge regression model (RRBLUP) through the “iPat” package (Chen and Zhang, 2018) was trained using whole genotype data (16,233 SNPs), and the AMP as the TP where BLUP for all environments and BLUP for Mansfield (MAN) and Waterville (WAT) site-years were used to calculate GEBV of each line belonging to the TST set of the previous year. Lines were ranked according to their GEBV and mean SMT scores. Scores of the top 50% selected based on BV and actual tolerance ratings were considered in comparing three different selection strategies-phenotypic selection (PS; lines selected based on SMT scores alone), GS (lines selected based on GEBV alone), and a combination of phenotypic and genomic selection (PS+GS; lines selected based on GEBV and SMT). Response to selection (*R*) was calculated using the formula *R = H^2^S* (Falconer and Mackay, 1996), where *H^2^* is the heritability value for snow mold for the year when GS model was trained; and *S* is the selection differential, calculated as *S* = µ_pop (with selection)_ - µ_pop(without selection)_.

## Results

### Snow Mold Tolerance Across Environments

**Figure 1:**
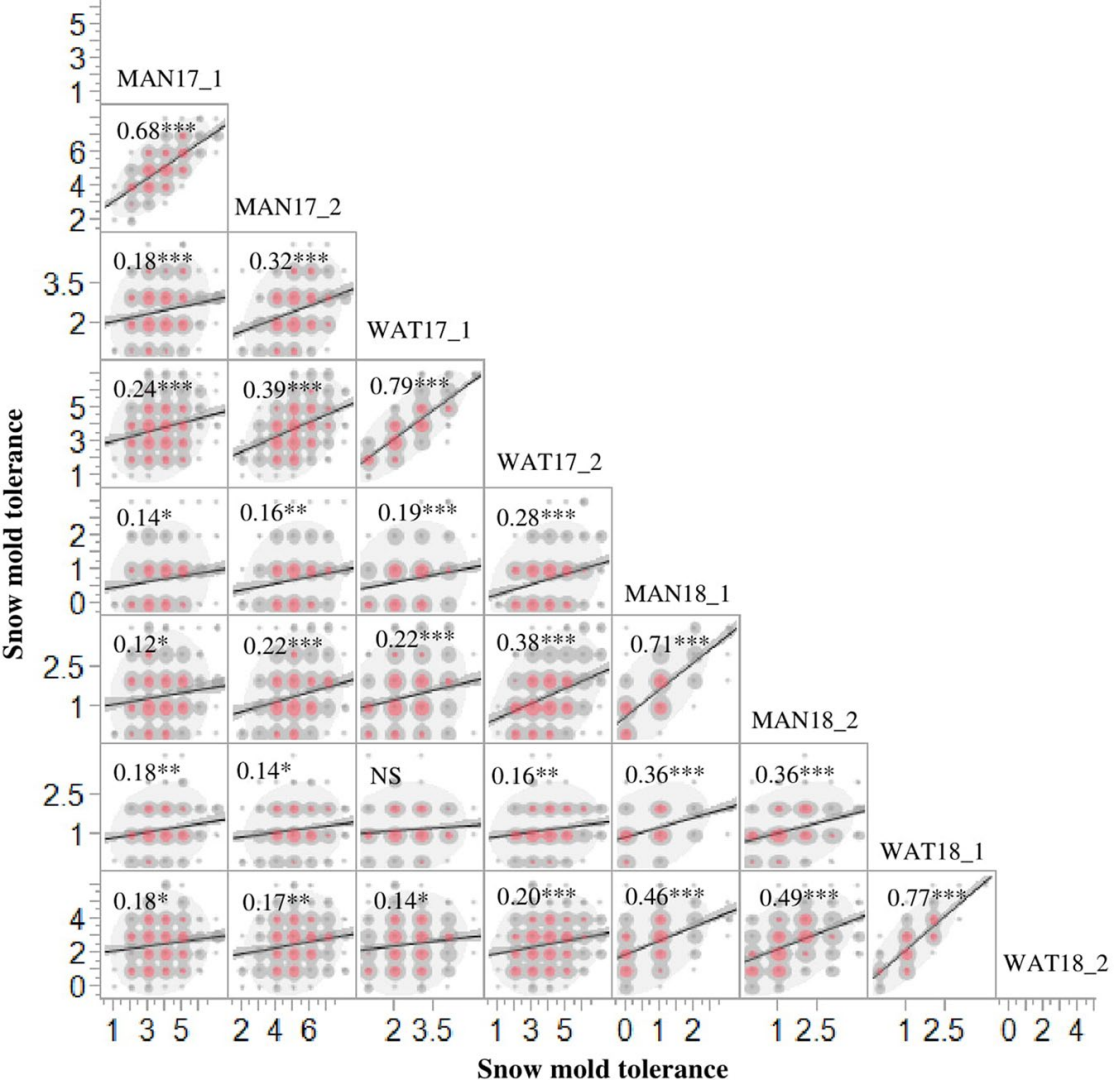
Phenotypic correlations for snow mold tolerance scores in Mansfield (MAN) and Waterville (WAT), WA in 2017 and 2018 for the association mapping panel (AMP). 1 and 2 indicate snow mold tolerance scores within a few days of snow melt and a month after snow melt, respectively. ***- significant at *P* < 0.0001; **- significant at *P* < 0.001; *-significant at *P* < 0.05; NS, Not significant.

Heritability (*H^2^*) values for snow mold tolerance varied across years and populations, ranging between 0.53 (2016) and 0.76 (2015) (**Table 1**). *H^2^* for tolerance to snow mold was 0.69 for the AMP. All environments for the AMP were significantly correlated, except for WAT17_1 and WAT18_1 (**Figure 1**). PCA showed clustering based on growing season (**Supplementary Figures 1A, B**), where PC1 caused 40.0 and 34.3% of variation for the AMP and the breeding lines, respectively. Disease pressure was greatest in 2017 (mean = 4.3) and 2018 (mean rating = 2.3) for the breeding lines. These ratings were slightly greater than the 2017 and 2018 mean snow mold scores for the AMP (3.8 and 1.5, respectively), indicating more general tolerance in the breeding lines than in the AMP. Mean scores for 2018 were significantly smaller (*P* < 0.0001) compared with the other years for both the AMP and the breeding lines, indicating that this year had the most favorable conditions for snow mold development. The tolerant variety Eltan had a mean disease rating of 3.6, whereas the susceptible variety “Finch” had a mean tolerance score of 2.6 across years.

**Table 1:** Heritability and mean snow mold tolerance scores for Pacific Northwest (PNW) winter wheat association mapping panel (AMP) and breeding lines evaluated between 2015 and 2018.

Population	No. of environments	No. of individuals	*H^2^*^a^	Mean rating^b^	Standard deviation	Range
AMP	4	458	0.69	2.64	1.76	0-8
TST2015	2	77	0.76	4.81	2.56	0-8
TST2016	3	110	0.53	4.86	1.44	0-8
TST2017	3	68	0.56	4.39	1.61	0-8
TST2018	3	40	0.75	2.41	1.70	0-7

^a^Broad-sense heritability; ^b^Snow mold scores were taken by rating lines within a few days of snow melt and one month later using a 0 (completely dead, with abundance of snow mold) to 10 (thriving, no snow mold) scale.

### Population Structure and Linkage Disequilibrium

**Figure 2:**
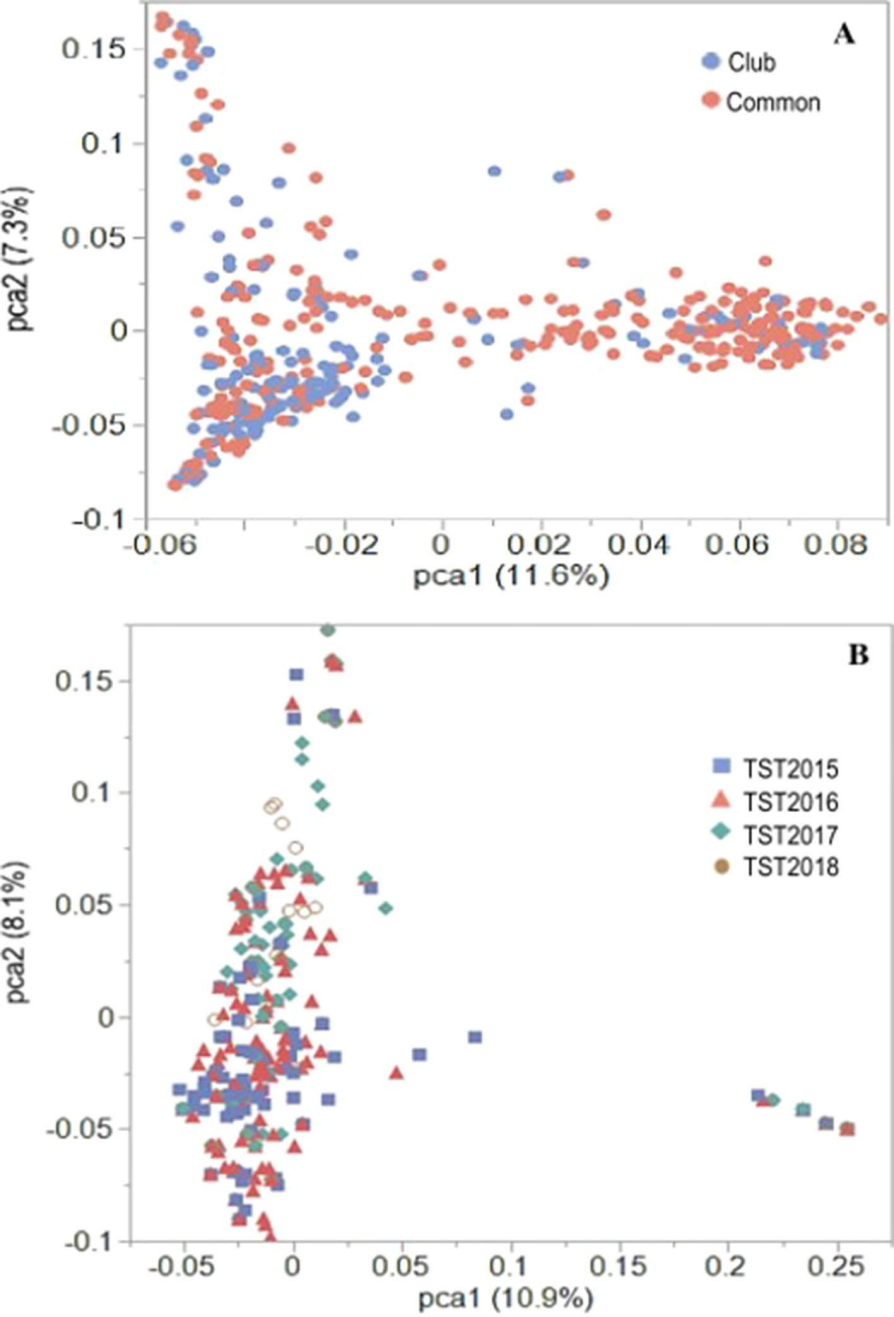
PCA plots of the first two principal components (PC) for the **(A)** US Pacific Northwest association mapping panel and **(B)** breeding lines using 16,233 GBS-derived SNP markers.

Previous PCA using Illumina 90K SNP marker data for the AMP showed distinct population stratification based on head-types (lax vs. club) (Jernigan et al., 2018). The reported *F_st_* value of 0.31 (Jernigan et al., 2018) supported the genetic differentiation between the market subclasses of wheat in the AMP. In the present study, PCA using 16,233 GBS markers confirmed this genetic structure for the AMP, although some overlaps within clusters of common and club wheat were still observed (**Figure 2A**). Genetic (Rogers) distance between the common and the club wheat for the AMP was 0.04, indicating a close relationship between the subclasses, and thus overlaps were expected. PC1 explained 11.6% of the phenotypic variation, whereas the PC2 explained 7.3% of the variation. Rogers distances of the breeding lines with the AMP ranged between 0.30 (TST2017 and TST2018) and 0.31 (TST2015 and TST2016), whereas *F_st_* coefficients were 0.23 (TST2016, TST2017, TST2018) and 0.24 (TST2015). PCA using GBS markers within the breeding lines described 10.9% and 8.1% of the variation caused by PC1 and PC2, respectively. Lines common to both the TP and TST sets formed a second cluster in the PC plot (**Figure 2B**). Prior analyses revealed a rapid LD decay over distance for this panel (estimated to be at ∼5 cM; Jernigan et al., 2018).

### Significant Marker-Trait Associations Identified

**Figure 3:**
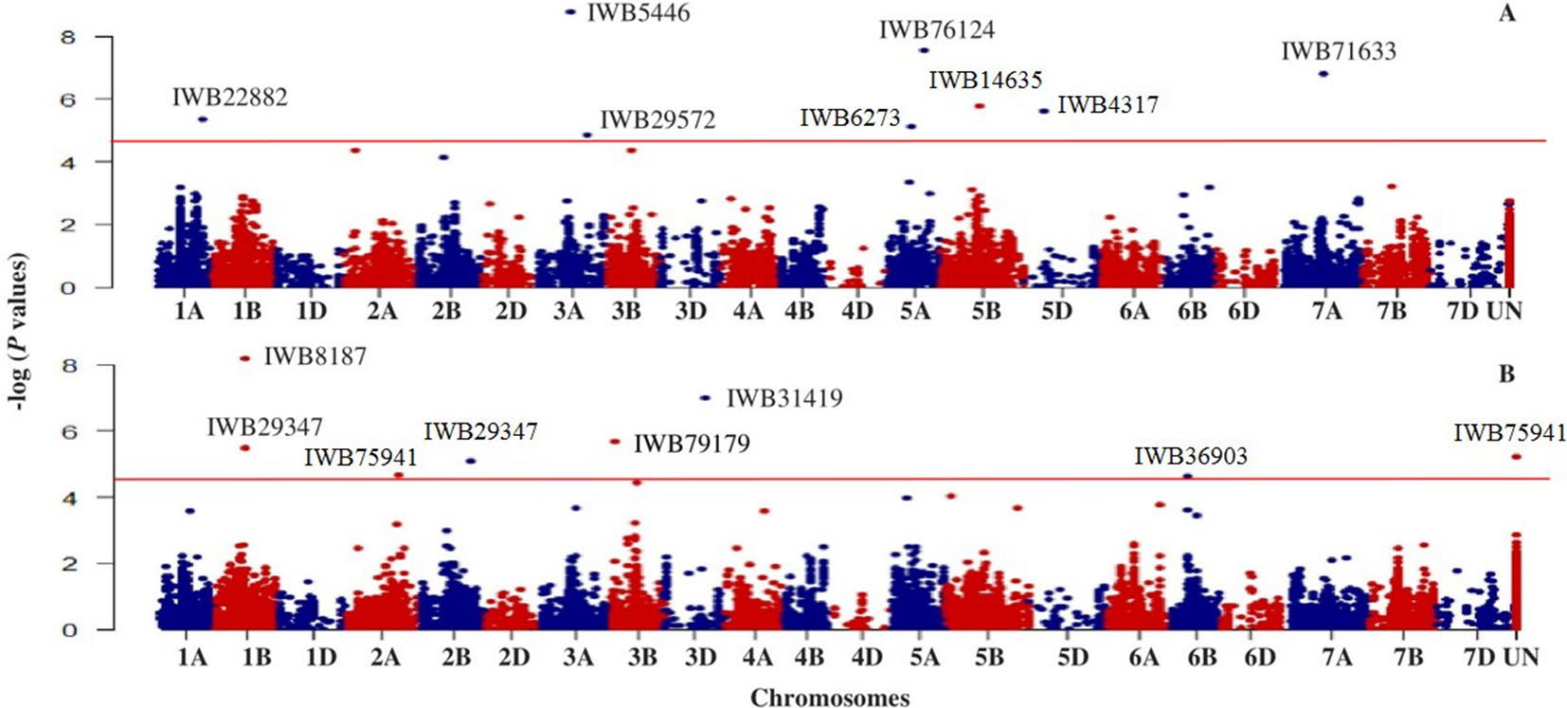
Manhattan plot showing genome-wide SNPs associated with tolerance to snow mold in Pacific Northwest winter wheat lines, BLUP for all environments; **(A)** no PC (PC0) and **(B)** using the first three PCs (PC3) in the GWAS model. Horizonal lines represent threshold for significance under a False Discovery Rate (FDR) of 0.05.

Based on GWAS, 100 significant markers (FDR = 0.05) were identified and distributed on 17 of 21 chromosomes (excluding 1D, 2D, 4D, and 6D), with percent of trait variation explained (PVE) ranging from 0.01 to 11.6% (**Supplementary Table 1**). **Figure 3** shows some of these significant SNPs for snow mold tolerance identified using BLUPs for all environments with the first three principal components included in the model (PC3) or without PC included in the GWAS model (PC0). Significant SNPs with unknown chromosomal positions totaled to 10, with PVE ranging between 0.07 and 5.70%. There were less deviations observed in the QQ plots when PCs were fitted as fixed effect in the GWAS models. Using only the first PC (PC1) for GWAS had the least deviations in the QQ plots in identifying significant associations in three (MAN, MAN17, and WAT18) out of seven environments used for GWAS.

Chromosome 5A had the greatest number of significant marker-trait associations (14), followed by 1B (9), 3B (9), and 1A (8) (**Supplementary Table 1**). The significant SNPs in 5A explained 0.10–11.6% of phenotypic variation, with additive effect ranging between −0.18 and 0.23, whereas the 1B SNPs were responsible for 0.01–8.4% of variation for snow mold tolerance (additive effect = −0.28–0.26). There were 114 intrachromosomal marker pairs in significant LD (*P* < 0.05) across all chromosomes and GWAS models. On chromosome 5A alone, there were 44 marker pairs (mean distance = 3.2 cM) in significant LD with an average *r^2^* of 0.12. Large haplotype blocks of significant markers in LD were observed on chromosomes 1A, 3A, 3B, 5A, and 5B. Significant SNPs on chromosomes 5A, 5D, and 7A were all in LD with at least one marker in the same chromosome. A total of 31 SNPs distributed on 15 chromosomes were considered stable (i.e., identified in at least two environments and/or GWAS model). Out of this number, five loci, *IWB36501* (3B), *IWB3779* (3B), *IWB65663* (5A), *IWB14635* (5B), and *IWB59690* (5B), were identified across all GWAS models used and responsible for 0.02–8.4% of phenotypic variation (**Table 2**). An unmapped locus, *IWB66039* (*R^2^* = 1.5-2.03%) was detected in two environments. GWAS using the GBS-derived SNPs also identified significant associations with tolerance to snow mold in similar chromosomes (data not shown).

**Table 2:** Single nucleotide polymorphism (SNP) markers associated with tolerance to snow mold across different models and/or environments in an association mapping panel of Pacific Northwest winter wheat lines.

SNP ID	SNP Name	Chromosome	Allele	Position (cM)	Minor allele frequency	Model^a^	Environment	*P*-value	Allele effect	*R^2^*^b^
*IWB36501*	*Jagger_c2707_152*	3B	A/**G**^c^	65.71	0.10	PC0	MAN18	1.046E-08	0.25	0.07
						PC1	MAN18	2.587E-08	0.24	0.06
						PC2	MAN18	1.622E-07	0.20	0.03
						PC3	MAN18	2.715E-06	0.18	0.08
*IWB3779*	*BobWhite_c5276_631*	3B	A/**G**	122.52	0.45	PC0	WAT17	4.216E-10	0.25	0.05
						PC1	WAT17	2.305E-10	0.26	0.05
						PC2	WAT17	1.377E-07	0.21	0.04
						PC3	WAT17	6.211E-07	0.21	0.00
*IWB65663*	*TA003225_1427*	5A	T/**C**	45.08	0.07	PC0	WAT	6.822E-06	0.16	0.05
						PC1	ALL	1.428E-06	0.16	0.04
						PC3	WAT18	5.246E-06	0.23	0.05
*IWB14635*	*CAP8_c2687_128*	5B	**A**/G	104.23	0.10	PC0	ALL	1.664E-06	0.16	0.04
						PC1	ALL	2.436E-06	0.17	0.04
						PC2	ALL	3.471E-06	0.15	0.00
						PC3	WAT	5.428E-07	0.18	0.00
*IWB59690*	*RAC875_c62460_650*	5B	A/**C**	188.58	0.14	PC0	MAN17	6.499E-06	0.22	0.06
						PC1	MAN17	1.574E-07	0.25	0.05
						PC2	MAN	4.308E-06	0.12	0.01
						PC3	MAN17	1.843E-08	0.30	0.05
						PC3	MAN	7.98E-10	0.16	0.01

^a^Number indicates the number of PC included in the GWAS model; ^b^Phenotypic variation explained by the SNP; ^c^Underlined allele is the minor allele; bold allele is the tolerant allele.

### Effect of Combining Favorable Alleles on Snow Mold Tolerance

**Figure 4:**
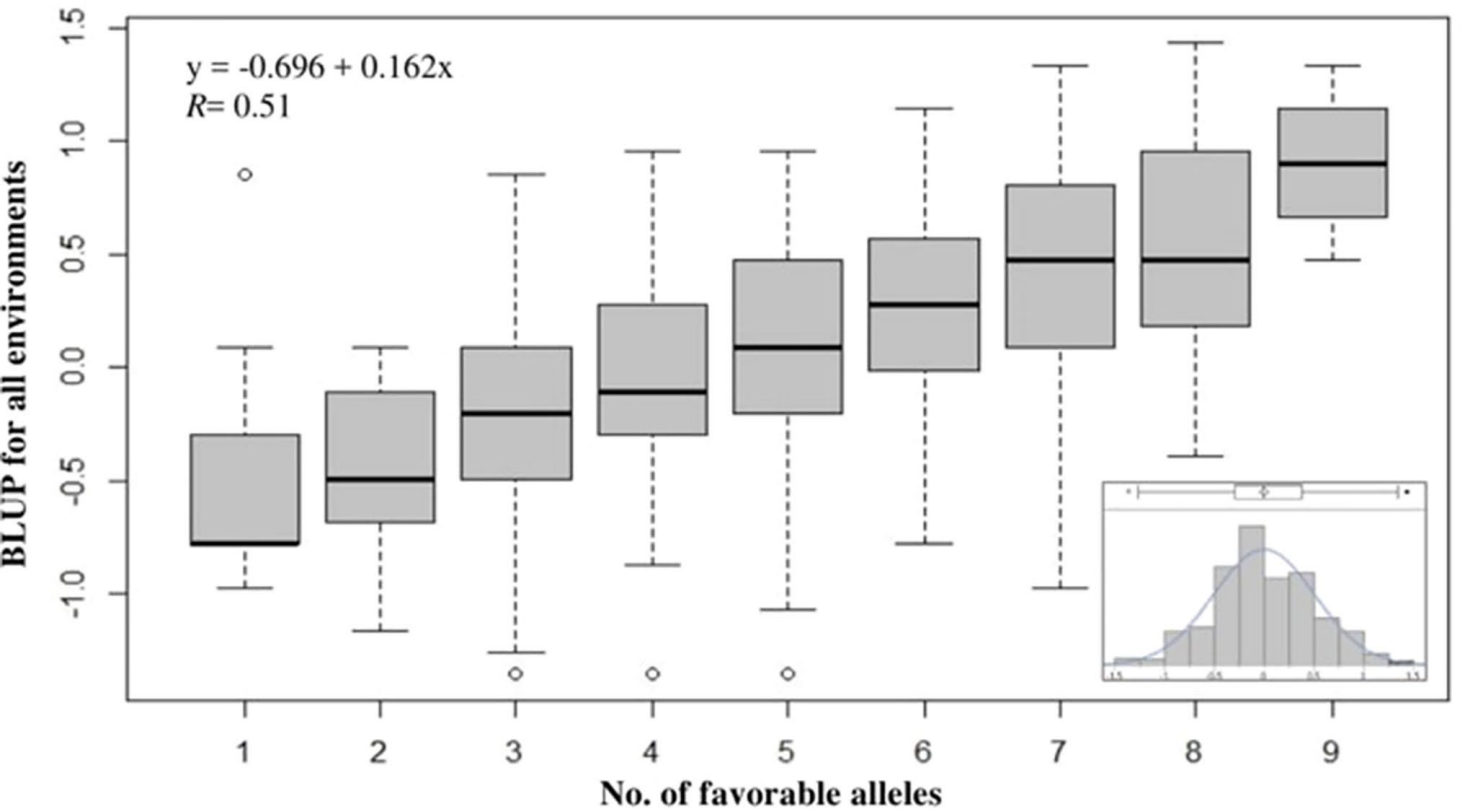
Effect of pyramiding favorable alleles for the 10 most significant SNP markers associated with tolerance to snow mold for the association mapping panel (AMP). Distribution of snow mold tolerance BLUP scores across all environments, AMP (inset).

To evaluate the cumulative effects of combining favorable alleles on snow mold tolerance in the AMP, BLUP values across all environments were regressed against the number of favorable alleles (ranged between 1 and 9) for 10 of the most significant SNPs associated with disease. These 10 SNPs (*P* value = 8.62E-11 to 2.73E-08) were distributed across eight chromosomes and responsible for 0.03–8.4% of phenotypic variation. Significant (*P* < 0.0001) differences among the groups having different numbers of favorable alleles were observed. Overall, a greater number of alleles with positive effects resulted in improved tolerance to snow mold (**Figure 4**; **Supplementary Table 2**). Mean comparisons among different groups having only allele differences at a single locus showed the same level of tolerance (e.g., mean tolerance of lines with eight favorable alleles vs. tolerance of lines having nine). Four lines, namely, “J90055-4”, “J90057-1”, “J970057-5”, and “WA8092”, contained nine favorable alleles (mean BLUP of 0.90), whereas 13 lines carried only a single favorable allele (mean BLUP of -0.49). The tolerant variety Eltan (BLUP = 0.76) had eight favorable alleles, whereas the susceptible variety Finch (BLUP = -0.01) had four. BLUP scores across all environments followed a normal distribution (**Figure 4**, inset).

### Independent Validations Using Winter Wheat Breeding Lines

Mean accuracy across all GS models, marker sets (MS), and TST populations was 0.36 (**Table 3**; **Supplementary Table 3**). There were no significant differences in predicting the breeding lines across years, and mean accuracy ranged between 0.29 (TST2018) and 0.41 (TST2015). Likewise, using different MS yielded similar mean accuracies: 0.39 for whole genotype data (MS1) and LD-tag SNPs (MS3) and 0.29 for significant SNPs (MS2). Mean accuracy for RKHS (0.84) was highest among the prediction models, whereas GBLUP and RRBLUP resulted to similar accuracies (0.11). Using BLUP for all environments for the TP to predict tolerance in individual locations for the TST lines did not show advantage over using only single environments for predictions. No significant differences were observed for predictions within the same environments, compared to across locations for different years.

**Table 3:** Accuracy of genomic selection for tolerance to snow mold across different models and marker sets (MS) using a Pacific Northwest association mapping panel (*N* = 458) as training set.

Population	Genetic distance^a^	GBLUP			RRBLUP			RKHS		
		MS1^b^	MS2	MS3	MS1	MS2	MS3	MS1	MS2	MS3
TST2015	0.31	0.21 (0.006)	0.22 (0.007)	0.19 (0.02)	0.22 (0.004)	0.22 (0.005)	0.18 (0.02)	0.87 (0.002)	0.87 (0.001)	0.75 (0.001)
TST2016	0.31	0.07 (0.08)	0.10 (0.11)	0.09 (0.08)	0.2 (0.11)	0.16 (0.10)	0.13 (0.04)	0.83 (0.03)	0.83 (0.03)	0.69 (0.04)
TST2017	0.30	0.21 (0.08)	0.18 (0.07)	0.004 (0.06)	0.19 (0.07)	0.18 (0.06)	0.006 (0.07)	0.90 (0.02)	0.90 (0.02)	0.81 (0.03)
TST2018	0.30	0.08 (0.04)	0.04 (0.07)	-0.09 (0.07)	0.006 (0.07)	0.03 (0.08)	-0.17 (0.06)	0.92 (0.03)	0.92 (0.03)	0.84 (0.04)

Standard deviations are in parentheses. ^a^Rogers distance with the panel; ^b^MS1- whole genotype data; MS2- significant (P < 0.05) SNPs identified from GWAS; MS3- LD-tag derived SNP markers.

### Correlation Between Genomic Estimated Breeding Values and Snow Mold Scores and Response to Selection

**Figure 5:**
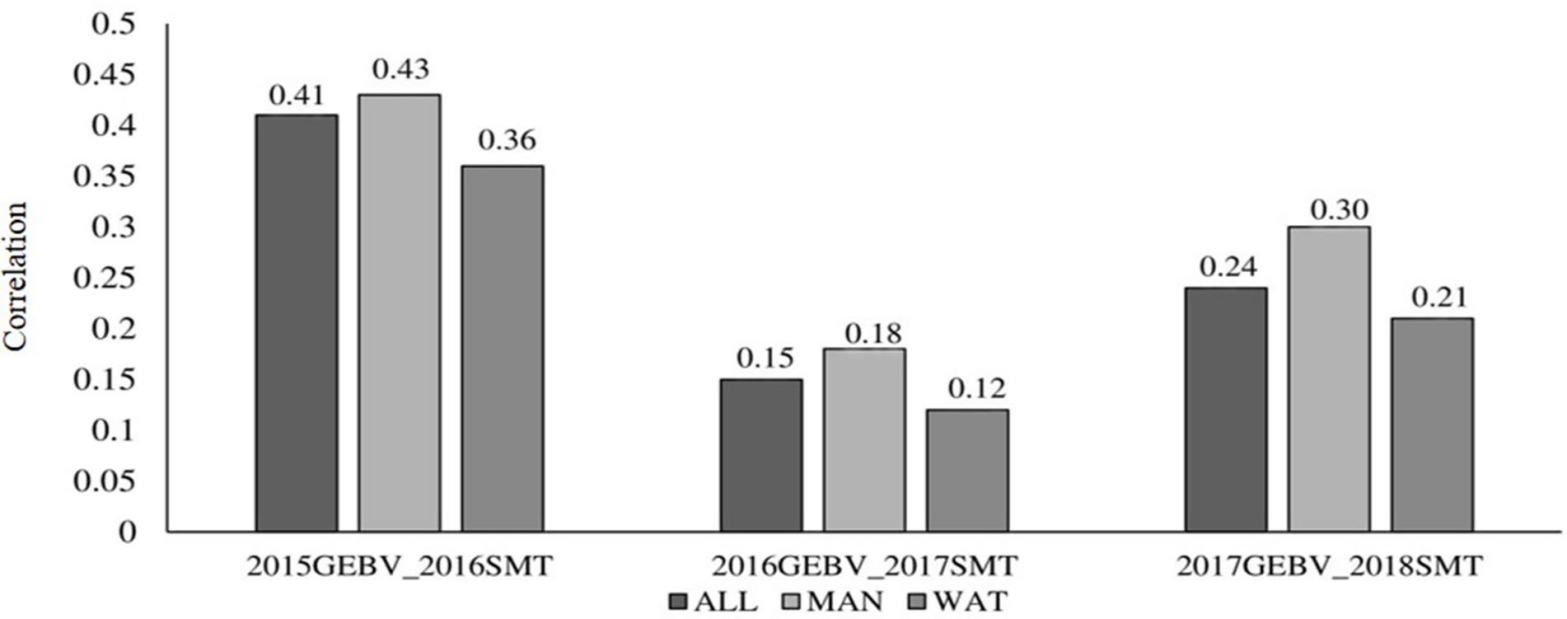
Correlation between breeding values of lines evaluated in a year and the mean snow mold tolerance (SMT) score observed for the following year. Datasets used to train the GS model are BLUP values for Mansfield and Waterville site-years (ALL), BLUP for Mansfield (MAN), and BLUP for Waterville (WAT) environments.

Correlation between GEBV calculated on a given year and snow mold ratings in the following year was low to moderate, ranging between 0.12 (2016–2017; BLUP for WAT) and 0.43 (2015–2016; BLUP for MAN) (**Figure 5**). Significant differences (*P* < 0.05) among mean correlations for each pair of years was observed, where 2015–2016 had the greatest correlations (mean of 0.40). In general, response to selection (*R*) was highest for PS across datasets, except for when PS+GS was used to select lines in 2016–2017 and 2017–2018 using BLUP values for all environments and BLUP for MAN as TP data, respectively (**Table 4**). Combining phenotyping with GS in these scenarios resulted in a 7 and 40% advantage in terms of *R*, compared to using PS alone. Among selection strategies, GS had the lowest values for response, with negative *R* in three datasets: BLUP for all environments (−0.04); BLUP for WAT site-years (−0.005) for 2015–2016; and BLUP for WAT site-years (−0.02) for 2017–2018. Applying a GS strategy, on average, resulted to a 0.05 increase in snow mold tolerance scores, whereas using PS+GS led to an increase of 0.59, on average, across datasets. Varieties Bruehl and Eltan consistently showed high BV and snow mold scores across different years.

**Table 4:** Response to selection (*R*) for phenotypic, genomic, and phenotypic + genomic selection strategies for tolerance to snow mold.

Dataset^a^	Selection method	2015-2016	Selection differential^b^	Response to selection	2016-2017	Selection differential	Response to selection	2017-2018	Selection differential	Response to selection
ALL	Phenotypic selection (PS)	5.38	0.66	0.50	5.25	0.67	0.36	3.87	0.83	0.56
	Genomic selection (GS)	4.77	0.05	0.04	4.51	-0.07	-0.04	3.06	0.02	0.01
	PS+GS	5.35	0.63	0.48	5.0	0.42	0.22	3.92	0.88	0.60
	Population mean^c^	4.72			4.58			3.04		
	Heritability, *H^2d^*	0.76			0.53			0.56		
MAN	PS	5.38	0.66	0.50	5.25	0.67	0.36	3.87	0.83	0.46
	GS	4.94	0.21	0.16	4.59	0.01	0.01	3.08	0.04	0.02
	PS+GS	5.65	0.93	0.71	5.13	0.55	0.29	3.75	0.71	0.40
	Population mean	4.72			4.58			3.04		
	*H^2^*	0.76			0.53			0.56		
WAT	PS	5.38	0.66	0.50	5.25	0.67	0.36	3.87	0.83	0.56
	GS	4.99	0.27	0.21	4.57	-0.01	-0.005	3.01	-0.03	-0.02
	PS+GS	5.38	0.66	0.50	5.07	0.49	0.26	3.81	0.77	0.43
	Population mean	4.72			4.58			3.04		
	*H^2^*	0.76			0.53			0.56		

Phenotypic dataset used to train the association mapping panel for calculation of breeding values; equal to BLUP for Mansfield and Waterville (ALL); and BLUP for Mansfield (MAN), and Waterville (WAT) site-years; ^b^Calculated as Selection differential, S = µ_pop (with selection)_ - µ_pop(without selection)_; ^c^Mean of the population without selection; ^d^Broad-sense heritability for snow mold for the year when the GS model was trained.

## Discussion

The current study identified genomic regions associated with tolerance to snow mold in a diverse population of PNW winter wheat lines through a GWAS approach. Additionally, GS was implemented through independent validations using this diverse panel to predict snow mold tolerance of WSU winter wheat breeding lines. Accuracies across different prediction models and marker sets were evaluated. Lastly, response to selection among different selection strategies was compared.

### Genomic Regions Associated With Snow Mold Tolerance Coincided With Major Freezing Tolerance and Vernalization Loci

In agreement with the first objective of this study, we detected SNP markers associated with snow mold tolerance in PNW winter wheat. A total of 14 significant snow mold tolerance markers identified in this study were on chromosome 5A. This region represents a large haplotype block covering an ∼88 cM distance. Analyses further revealed significant (*P* < 0.0001) associations of these haplotypes in 5A with snow mold tolerance, although no stable, large effect (*R^2^* > 10%) loci were identified in GWAS. Significant SNPs on chromosome 5A co-localized with *QTLSelt.wpg-5A.I* (*R^2^* = 47%) and *Fr-A2* (*R^2^* = 49%) previously identified to be associated with snow mold and freezing tolerance, respectively, in an Eltan x Finch biparental mapping population (Kruse et al., 2017). Based on a consensus map by Wang et al. (2014), the flanking marker *Xiwb53912* (59.28 cM) of these QTL regions is located near *IWB23857* (64.21 cM), identified to be associated with snow mold tolerance in this study. Two other significant markers, namely, *IWB76844* and *IWB25201*, on 5A coincided with the major vernalization gene *Vrn-A1*. Significant SNPs on chromosome 5B formed a large haplotype block covering a ∼150 cM distance, near *Fr-B1* (Tóth et al., 2003) and *Vrn-B1*, although no significant association between these haplotypes and snow mold tolerance was observed in the current study.

Our results and those of a previous study (Kruse et al., 2017) showed that loci affecting both snow mold and freezing tolerance are in similar genomic regions, indicating potential pleiotropic effects or tight linkage. Tolerance to cold might result from a more effective fungal resistance mechanism due to pleiotropy, as reduced cold damage may result in heathier plants that can resist fungal infections (Kruse et al., 2017). Lines from the AMP observed to have high tolerance to snow mold also possessed cold tolerance alleles on three C-repeat binding factor (CBF) loci (S Carle, unpublished data), suggesting that selecting lines that can survive cold temperatures could also result in selection for lines with snow mold tolerance. Major low-temperature tolerance QTL were previously identified in chromosome 5A (Båga et al., 2007; Case et al., 2014) and 5B (Zhao et al., 2013) near known freezing tolerance and vernalization genes, showing the relevance of these loci in winter survival, as well as tolerance to fungal diseases related with low temperature conditions.

Snow mold tolerance markers other than those related with major cold tolerance and vernalization genes were identified on chromosomes 4B and 6B, similar to results previously reported *via* biparental mapping in winter wheat (Kruse et al., 2017). SNP *IWB12434* (4B; 74.62 cM) co-located with *IWA3240* (65.28 cM) and *IWA908* (79.01 cM), markers flanking the freezing tolerance QTL, *QFfin.wpg-4B*, whereas significant SNPs in 6B, namely, *IWB33837* (73.41 cM) and *IWB33838* (116.24 cM) were mapped near the *QSfin.wpg-6B* locus (flanked by *IWB7981*; 93.50 cM) associated with snow mold tolerance (Kruse et al., 2017). Overall, our results demonstrated the potential of GWAS as a complementary mapping tool in dissecting genomic regions for tolerance to snow mold. SNP markers on chromosomes other than 4B, 5A, and 6B identified here have not been reported elsewhere, and hence represent novel SNP markers affecting snow mold tolerance in PNW winter wheat.

### Combining Favorable Alleles From Significant Loci Is Related to Improved Snow Mold Tolerance

Regression analyses showed the direct relationship between increasing number of favorable alleles and increased snow mold tolerance demonstrating an additive response. Previously, pyramiding favorable alleles was observed to increase resistance for stripe rust (Naruoka et al., 2015) and eyespot disease (Lewien et al., 2018) for the same population of US PNW winter wheat lines. Lines differing at a single locus (e.g., lines with eight favorable alleles vs. lines having nine) had similar levels of tolerance, suggesting that additive effects for these alleles might not be enough for significant differences to occur. Moreover, it was observed that some lines having negative BLUP scores for tolerance have as many as seven or eight favorable alleles, further demonstrating the genetic complexity of snow mold tolerance. Likewise, Emebiri et al. (2017) observed that some wheat lines having alleles for Sunn pest (*Eurygaster integriceps* Puton) resistance were phenotypically susceptible to the disease, indicating weak genotype-phenotype associations. The absence of strong associations between alleles and phenotypes could be a consequence of different evolutionary processes and the large number of generations in plant breeding (Ellis et al., 2007). It also could be the effect of multiple pathways within the plant contributing to tolerance to snow mold (like the demonstrated association with cold tolerance). The lack of stable, large-effect loci could limit the efficient implementation of marker-assisted selection (MAS) for snow mold tolerance, and, hence, GS is seen as a good complement to GWAS. Lines having favorable alleles for the significant loci identified here represent novel sources of tolerance in PNW winter wheat lines.

### Relatedness Is a Key Player in Genomic Selection for Snow Mold Tolerance

The fact that snow mold is affected by many loci with small effects, as revealed through GWAS, makes it an ideal trait for genomic selection. GS is an alternative solution for dissecting genetic architecture of complex traits due to the limitation of MAS in identifying small effect loci (Mirdita et al., 2015). In this study, a diverse population of winter wheat lines was used as the training panel to predict snow mold tolerance in WSU winter wheat breeding lines evaluated between 2015 and 2018. Accuracy values were assessed across different marker sets (whole genotype data, significant markers, and LD-tag SNPs) and models (RRBLUP, GBLUP, and RKHS).

High relatedness between TP and validation sets has been associated with increased GS accuracies (Asoro et al., 2011; Lorenz and Smith, 2015), whereas a lack thereof between populations resulted in decreased accuracies (Charmet et al., 2014). Limited genetic relatedness between the training and test lines reveals that the extent of LD is shorter and unstable across individuals in the population (Tan et al., 2017). RRBLUP relies mainly on the strength of LD between markers and QTL, where an increase in marker-QTL LD is expected to improve predictions (Lorenz and Nice, 2017). In our case, as there is no strong marker-QTL LD observed due to low relatedness between TP and validation sets, the implementation of the RRBLUP model for GS resulted in inter-year prediction accuracies close to zero. RRBLUP also performed poorly compared with its Bayesian counterparts in predicting flowering time and grain number using unrelated double haploid populations of wheat (Thavamanikumar et al., 2015). Similarly, the GBLUP model, which depends mainly on the genomic relationships between the training and selection set (Lorenz and Nice, 2017), had low accuracies, most likely also a consequence of the genetic relationships between the training and test populations. Low relatedness between the TP and selection candidates further suggests the presence of opposite linkage phases between markers and QTL (Haile et al., 2018), which negatively affects the accuracy of predictions.

The use of lower marker density panels presents a cost-efficient alternative to using whole genotype data for GS. In this study, however, implementing LD-tag SNPs and GWAS-derived markers did not lead to a significant decrease in prediction accuracy, where, in most scenarios, using these subsets typically caused a reduction in accuracies. Likewise, Juliana et al. (2018) observed loss in accuracy when marker subsets were used for predicting yield across unrelated CIMMYT elite yield trial nurseries. Without a close relatedness between the training and test candidates, it has been shown that higher marker numbers are needed for more accurate predictions in both empirical and simulation studies (Hickey et al., 2014; Norman et al., 2018). More markers are also required in a breeding scenario where older lines are used to predict newer germplasm (Rutkoski et al., 2017), as in our case, where the AMP was used to predict snow mold tolerance of WSU winter wheat breeding lines. In spring wheat, Muleta et al. (2017) observed that using all available SNP markers was necessary to reach the highest attainable accuracy for predicting stripe rust resistance in a diversity panel. The rapid decay of LD in the TP used (Jernigan et al., 2018) further demonstrates that more markers are necessary for achieving accurate predictions. A greater number of markers are needed to train a model for GS when LD decays rapidly (Poland and Rutkoski, 2016). Conversely, when LD decay is slow for a population, implementing a subset of SNPs for GS was enough to achieve similar accuracies with that of a full set of markers (Cericola et al., 2017). Removing SNPs potentially decreased the number of marker-QTL in LD captured, leading to lower accuracies. Our results support the relevance of genetic relatedness between training and validation populations in achieving more accurate predictions, particularly for GS models that rely on LD between QTL and markers for predictions.

### Modeling Nonadditive Effects Improved Prediction Accuracy in the Absence of Close Relatedness Between Training and Validation Populations

Previous studies in wheat focused on cross-validations (Heffner et al., 2011; Heslot et al., 2012), where a single population is partitioned into training and testing sets. More recently, independent validations that use different populations as training and validation sets were implemented in wheat (Thavamanikumar et al., 2015; Haile et al., 2018). The potential of independent predictions for snow mold tolerance was demonstrated across different models and marker sets used, even without a close relationship between the training panel and validation sets.

Although there was no close relatedness between the training and test populations based on genetic (Rogers) distances, we observed that modeling nonadditive effects can lead to improved predictions. RKHS model showed superiority compared to GBLUP and RRBLUP for all marker and datasets used. Our results were consistent with previous reports in wheat when RKHS model was used for predicting grain yield (He et al., 2016; Huang et al., 2016; Song et al., 2017), and Fusarium head blight and *Septoria tritici* blotch (Mirdita et al., 2015). When there were no lines common to both TP and validation sets, there was an increase in accuracy using RKHS (data not shown), which also suggests that this model is not affected by changes in population composition and relatedness. Nonparametric or semiparametric approaches such as RKHS are built to model complex and nonexplicit interactions, thereby maximizing predictive ability (Varona et al., 2018). In accordance with our findings, the use of GS models that can capture nonadditive genetic effects to achieve increased accuracies is thus recommended when evaluating populations with low genetic relationships.

### Complementing Traditional Phenotyping With Genomic Selection Shows Potential for Increasing Tolerance to Snow Mold in PNW Winter Wheat

Being the baseline method, PS was expected to have the greatest response to selection (*R*); nevertheless, we observed the potential for selecting lines with improved tolerance to snow mold by combining PS with GS, showing a maximum of 40% merit in *R* for the 2016 selections (0.71 for PS+GS vs. 0.50 for PS alone). Recently, Belamkar et al. (2018) observed that using both GEBV and phenotype data in preliminary yield trials increased opportunity to select better yielding lines across environments and years in comparison to those selected based on phenotype alone in a single year. Likewise, it has been shown that merging GS data based from multienvironment trials with PS in preliminary trials resulted to a much better performance in predicting yield than using either method alone (Michel et al., 2017). Line selection based exclusively on GEBV for snow mold tolerance had a disadvantage, as some lines with high GEBV on one year might not necessarily have high tolerance scores the following year. This was demonstrated by negative *R* values and the low correlations between GEBV and tolerance scores observed in some of the datasets evaluated. Therefore, caution is still warranted when relying on GEBV alone for selection decisions.

Increasing prediction accuracies by using a training set that fully captures genetic relatedness with the validation populations, choosing the appropriate GS model, and the number of markers for predictions in the presence/absence of marker-QTL LD, would make selections *via* GEBV more reliable. Regardless, it is still possible to increase tolerance by selecting superior lines based on breeding values alone despite having low to moderate correlations between GEBV and disease scores across years. In maize, the feasibility of a breeding program based on GS that resulted in greater genetic gain per year has been previously demonstrated under low to moderate prediction accuracies (Heffner et al., 2010). This further shows that prediction accuracies obtained are not a true measure of the success of implementing GS strategies in breeding programs (Belamkar et al., 2018) but more so how effectively GEBV can be used for selection decisions (Juliana et al., 2018). Overall, breeders should consider using both GEBV and phenotypic information when evaluating important disease-related traits, in choosing which lines to advance or in selecting parental lines for the breeding program.

## Conclusions

A genome-wide association study identified SNP markers associated with tolerance to snow mold in a diverse population of PNW winter wheat. Significant SNPs co-localized with QTL for freezing tolerance and vernalization genes demonstrate possible pleiotropic effects for low temperature and snow mold tolerance. A direct relationship between the number of positive alleles for significant loci and snow mold tolerance was observed. Implementing GS using independent sets of samples as validation populations showed the potential for improving tolerance to snow mold. Combining GS with phenotypic selection showed the possibility of increasing genetic gains by selecting lines with improved tolerance based on breeding values and actual snow mold ratings. Relatedness between the training and validation panels is important for achieving accurate predictions, and modeling nonadditive effects improved accuracies in the absence of close genetic relationships between populations. Altogether, results from GWAS and GS demonstrated the complex genetic architecture of tolerance to the disease. To the best of our knowledge, this is the first major study on the genetic dissection of snow mold tolerance using GWAS and GS approaches in PNW winter wheat lines.

## Author Contributions

DL analyzed data and drafted the manuscript. JG analyzed data and edited the manuscript. TM edited the manuscript. BW performed genotyping-by-sequencing variant calling and edited the manuscript. AC edited the manuscript and obtained funding for the project.

## Funding

The authors would like to thank the Washington State University (WSU) Hatch project #0232 and the National Institute of Food and Agriculture (NIFA) of the U.S. Department of Agriculture, under award number 2011-68002-30029 and 2016-68004-24770 for funding.

## Conflict of Interest

The authors declare that the research was conducted in the absence of any commercial or financial relationships that could be construed as a potential conflict of interest.
