# Supplementary material for: Genetic Dissection of Snow Mold Tolerance in US Pacific Northwest Winter Wheat Through Genome-Wide Association Study and Genomic Selection
Source: Front Plant Sci. 2019 Oct 29;10:1337. doi: 10.3389/fpls.2019.01337 (PMC6830427; doi:10.3389/fpls.2019.01337)
Supplement: Supplementary file 1 [file Image_1.pdf]

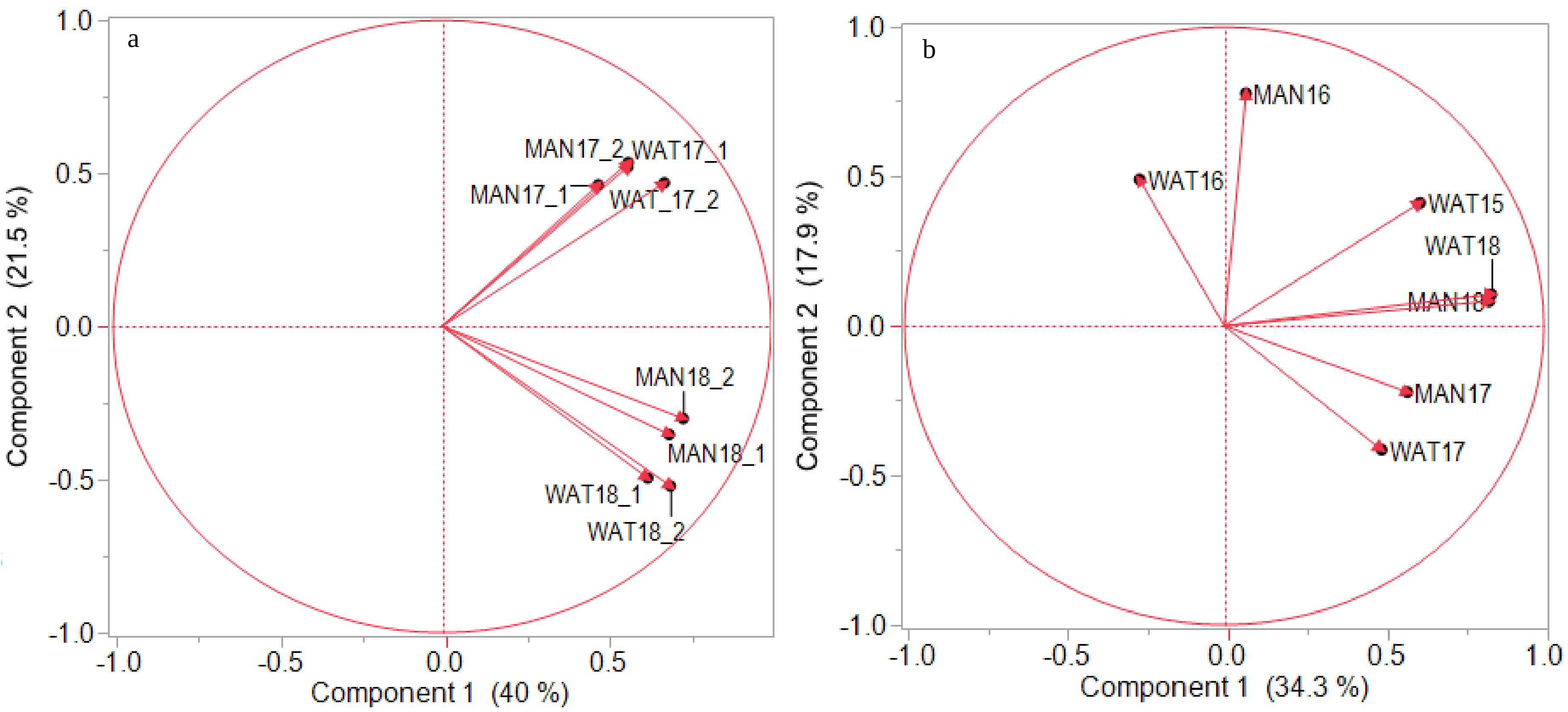

Supplementary Fig. 1. PCA bi-plots for tolerance to snow mold across environments in Mansfield and Waterville, WA for the association mapping panel (a) and Washington State University (WSU) winter wheat breeding lines (b)
